# Megavoltage photon FLASH for preclinical experiments

**DOI:** 10.1002/mp.17891

**Published:** 2025-05-19

**Authors:** Edward R. J. F. Taylor, Iain D. C. Tullis, Borivoj Vojnovic, Kristoffer Petersson

**Affiliations:** ^1^ Department of Oncology University of Oxford Oxford UK; ^2^ Radiation Physics Department of Haematology Oncology and Radiation Physics Skåne University Hospital Lund University Lund Sweden

**Keywords:** bremsstrahlung, FLASH, linac, megavoltage, photon, tungsten

## Abstract

**Background:**

FLASH radiotherapy using megavoltage (MV) photon beams should enable greater therapeutic efficacy, target deep seated tumors, and provide insights into mechanisms within FLASH.

**Purpose:**

In this study, we aim to show how to facilitate ultra‐high dose rates (FLASH) with MV photons over a field size of 12–15 mm, using a 6 MeV (nominal) preclinical electron linear accelerator (linac). Our intention is to utilize this setup to deliver FLASH with MV photons in future preclinical experiments.

**Methods:**

An electron linear accelerator operating at a pulse repetition frequency of 300 Hz, a tungsten target, and a beam hardening filter were used, in conjunction with beam tuning and source‐to‐surface distance (SSD) reduction. Depth dose curves, beam profiles, and average dose rates were determined using EBT‐XD Gafchromic film, and an Advanced Markus ionization chamber was used to measure the photon charge output.

**Results:**

A 0.55 mm thick tungsten target, in combination with a 6 mm thick copper hardening filter were found to produce photon FLASH dose rates, with minimal electron contamination, delivering dose rates > 40 Gy/s over fields of 12–15 mm. Beam flatness and symmetry were comparable in horizontal and vertical planes.

**Conclusion:**

Ultra‐high average dose rate beams have been achieved with MV photons for preclinical irradiation fields, enabling future preclinical FLASH radiation experiments.

## 1 INTRODUCTION

FLASH radiotherapy utilizes a differential sparing effect between normal and tumor tissues to broaden the therapeutic window within the modality of external beam radiotherapy. While the most pertinent choice of temporal and spatial parameters remain to be determined, average ultra‐high dose rates (UHDR) of at least ∼30 Gy/s have been observed to induce this effect.^1^, ^2^ Because of the short delivery time, FLASH radiotherapy has the potential to increase patient throughput, reduce effects of anatomical movement, and reduce uncertainties in targeting tumor volumes,^3^, ^4^ in conjunction with appropriate low latency imaging techniques.

Radiotherapy is most commonly delivered with electron, proton, or photon beams. However, the clinical usefulness of electron beams is limited by their short penetration at clinically used energies of around 6–20 mega‐electron‐volts (MeV), making them appropriate to treatment of superficial sites. Proton (or ion) beam radiotherapy has the advantage of well‐defined irradiation volumes, achieved through the use of spot scanning and spread‐out Bragg peak energy deposition; however, this modality is reliant on access to highly complex, large, and expensive installations. As a result, < 1% of radiotherapy patients worldwide receive treatments with protons or heavier ions.^5^

Radiotherapy using megavoltage (MV) photon beams is the most common radiation modality by far, with ∼90% of cancer patients treated globally with radiotherapy,^5^ using increasingly sophisticated methods to treat targets of interest, such as intensity‐modulated techniques,^6^ or volumetric modulated arc therapy (VMAT)^7^. A standard clinical linear accelerator (linac) can treat with both electron and photon beams, though photon beams are preferred (in all but relatively rare superficial treatments) because of their ability to reach deep‐seated tumors, their sharper penumbra, and their robustness to variations in treated tissue density.

It is thus likely that MV photons will eventually be used for FLASH radiotherapy, if this is to become a commonplace clinical modality. However, the techniques used to achieve high conformality with MV photons rely on intensity‐modulation and/or delivery from multiple beam angles, both of which are difficult to achieve within FLASH timescales, as these require mechanical motion of the multileaf collimator (MLC) and gantry. To achieve FLASH dose rates, a ∼1000‐fold increase in average dose rate will be required, and approaches to reach this are underway.^8^ However, such FLASH treatments should ideally be delivered as with technologies comparable to those used by current installation, albeit with a higher peak output and reduced operational duty cycles. The conversion efficiency from electron beam power to photon power increases with increasing electron beam energy,^9^, ^10^ so high energies are suggested, though these should preferably not exceed 15–18 MeV, to minimize neutron activation of materials used in the construction of the irradiation facility.^11^

To date, the vast majority of preclinical studies of the FLASH effect have been performed with MeV electron beams. Clearly, FLASH radiation studies remain to be evaluated using photon irradiation prior to clinical deployment. Understanding the preclinical efficacy of MV photon, FLASH radiation is vital given that the accessibility and familiarity of MV photon beam treatments could preclude a swift global clinical translation for FLASH radiotherapy, unmatched by other treatment modalities. Previous FLASH studies have utilized synchrotron sources and lower energy x‐ray tubes, ^12^, ^13^ which have favorable characteristics compared to MeV electrons and MV photon beams such as a sharp penumbra and are now available in self‐shielded cabinets with on board image guidance.^14^

In this work, we present an approach that allows the use of a conventional linac to deliver FLASH photon beams suitable for preclinical research. Most clinical photon sources are able to deliver a flattening‐filter‐free (FFF) average dose rate of ∼0.2 Gy/s (12 Gy/min) at a source‐to‐surface distance (SSD) of ∼1 m.^15^ Calculations suggest that, assuming the inverse‐square law holds (despite a linac source not being a point source), an average dose rate of 80 Gy/s can be obtained at an SSD of 5 cm, allowing FLASH dose rates to be reached. However, the treatment area is also, of course, reduced. We show here that such a reduction of SSD can be exploited for preclinical irradiations, where field sizes are usually small. We describe the design of a suitable target that can be used as an “add‐on” to linacs used for preclinical FLASH irradiations using electrons.

Clinical linacs are designed to accelerate pulses of electrons onto a target that generates bremsstrahlung photons. The target, the electron energy, and the average beam current determine the photon beam dose rate (comprised of two components: the instantaneous dose rate —dose per pulse over pulse width, and average dose rate—total dose over total time) and photon energy spectrum. The average beam current is governed by the electron (macro)pulse peak current and the electron pulse repetition rate.^16^ In clinical linacs, the power deposited in the target is of the order of 10^3 ^W and the photon‐generating targets are designed to run continuously, requiring appropriate target cooling. The highest photon dose rate output is ultimately determined by the peak radiofrequency power used to accelerate the electrons, by the ability to inject electrons into the accelerating waveguide structure, and by the design of the structure and by its shunt impedance.^16^ The target is usually placed within the accelerator's vacuum system to eliminate the possibility of target oxidation. Although the efficiency of photon production increases with electron energy, our linac is limited to only produce 6 MeV (nominal) beams. The overall target thickness is usually made to stop all incident electrons and it is well known that composite Tungsten‐Copper targets are preferred when high photon outputs are required.^17^, ^18^

Tungsten is commonly used as the target material, due to its high electron density and high melting point. Tungsten targets are usually backed by a thick layer of copper or other thermally conducting, water‐cooled material. This backing material removes electron contamination of the resulting beam but can limit the maximum dose rate achievable, as its temperature limit is reached earlier than that of the tungsten. Moreover, dissimilar expansion at contact interfaces ^19^, ^20^ is inevitable. The use of thick backing materials results in higher photon beam attenuation and lower temperature fluctuations during the macropulse temporal profile but harder photon beams are generated. The penetration of energetic electrons in conducting solids is usually defined by the continuous slowing down approximation (CSDA) of the highest energy of the incident electrons.

When a combination of materials is used for targets, the condition described in Equation 1 is fulfilled.^21^, ^22^ Because the ${R_{CSDA}}_{copper}$ ∼4.5 mm at 6 MeV (${R_{CSDA}}_{copper}$∼5.3 mm at 8 MeV) a 6 mm copper backer alone, ignoring any electron energy loss in the tungsten or in air, can be considered more than enough to minimize electron surface dose contamination.^22^, ^23^

(1)

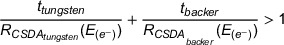




The CSDA range is known to overestimate the electron range as it assumes a straight line trajectory, though with Ebert's formulation from Tabato, Iko, and Okabe, we still provide a combination of materials that exceeds the extrapolated range of electrons (for copper and tungsten at 6 MeV, these are 2.9 and 1.1 mm, respectively).^24^, ^25^

Using a thin tungsten target not only minimizes self‐attenuation of photons generated, but also enables higher dose rate output as less energy is converted to heat, which enables the use of higher input currents.^19^ Owing to a very small heat diffusion length ^26^ (∼0.03 mm) in tungsten relative to the electron range ($R_{CSDA_{tungsten}}$∼2.21 mm at 6 MeV), ^10^ the temperature rise of the focal spot is said to be limited by the heat capacity, ^27^ such that the temperature rise is proportional to the incident power density and pulse length, and inversely proportional to the element mass (ρ ∼19,300 kg·m^−3^) and the heat capacity (c ∼133 J/kg/K).^26^ However, the applicability of these models, established for anode targets in x‐ray tubes, is limited for our case; where electron transport is non‐negligible with the position of maximum energy absorption at an appreciable depth (∼0.3 mm ^28^) in the target (akin to a volumetric heat source), ^27^ irradiations utilize a pulsed rather than continuous delivery, lateral heat redistribution occurs, and thermal radiation is significant for unpolished targets.^29^, ^30^ These models also highlight how increasing the focal spot size (e.g., achieved by moving the target some distance from the beam exit window) can reduce the incident power density, temperature increase in the focus, and increase the target lifetime without a substantial reduction in photon dose rate.^22^ It is noted that the duty cycle of preclinical FLASH irradiations is, in general, relatively low and the irradiation time is short (< 1 s). Target cooling at short times can thus be simplified for preclinical work, to the extent that the target can be placed outside the accelerator vacuum system.

The aim of this study is to show beamline modifications resulting in a preclinical radiation platform that can be used to monitor and deliver FLASH with MV photons.

## 2 METHODS

### 2.1 Linac

The accelerator uses an Elekta SL75 travelling wave waveguide and S‐band radiofrequency magnetron (Teledyne e2v‐M5125 type); this was used for a pulsed beam delivery (akin to clinical linacs), at a pulse repetition rate of 300 Hz, delivering 3.4 µs wide macropulses at peak currents of ∼100 mA and a duty cycle of just over 0.1% (Table 1). The “beam on” time was restricted to 1 s. The accelerator installation features a device to monitor the electron beam energy, ^31^ used here only during accelerator set up, and a non‐intercepting electron beam charge monitor (toroidal inductive sensor), ^32^ used to integrate the total charge delivered and can be used to shut off triggering of the accelerator once the required charge has been delivered.

**TABLE 1:** Linear accelerator characteristics.

Linac structure	Travelling wave type
Nominal electron energy	6 MeV
Resonant frequency	2.998 GHz
RF peak input power	2 MW
Pulse length	3.4 µs
Pulse repetition rate	25–300 Hz
Peak pulse current	∼100 mA
Average pulse current	∼0.1 mA
Maximum beam power	∼700 W

### 2.2 Target construction

**FIGURE 1:**
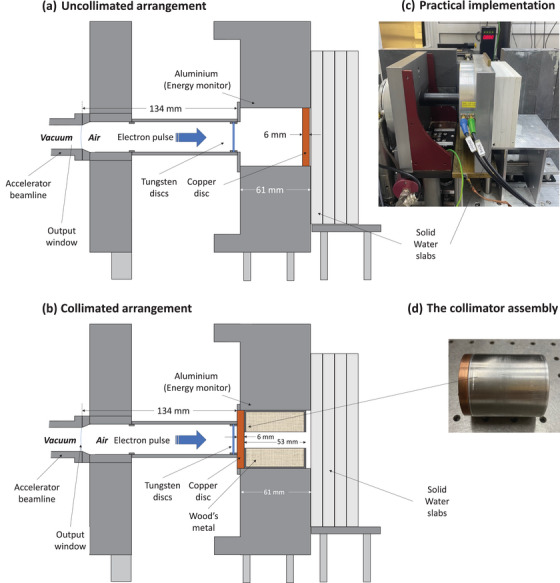
Uncollimated (a) and collimated (b) bremsstrahlung target assemblies, drawn to scale, showing the electron beam impacting a series of tungsten discs. The large aluminum structure is an electron beam energy monitor as described in Berne et al.^31^; it is not used when the target materials are inserted but forms a convenient support structure. Solid water slabs (150 × 150 mm^2^) cross‐section, of varying thicknesses are placed downstream (SSD ≥ 64 mm) and enable EBT‐XD film and Advanced Markus ionization chamber charge output measurements to be performed at any given depth in the solid water. In the case of the collimated arrangement, a 14 mm aperture allows passage of photons. Panel (c) Practical arrangement of the target assembly. Panel (d) Image of the collimator.

Our in‐house developed preclinical FLASH‐optimized 6 MeV (nominal) electron linac produces an approximately circular electron beam of ∼5 mm diameter full width at half maximum (FWHM) as it exits through the 10 µm thick beryllium‐copper beamline exit window.^31^ The exit window marginally influences the electron energy spectrum and scatters electrons, which also occur in the air‐filled region between the window and target as shown in Supplementary appendix (Figure S1). A hollow cylindrical aluminum tube was placed directly downstream of the exit window and varying numbers of tungsten discs were placed as a bremsstrahlung target at the end of the tube, held in place by two retaining rings. The beam diameter at target location is ∼10 mm (FWHM) (Figure S2). Two arrangements were investigated, as outlined in Figure 1. The first arrangement uses a copper disc some distance away from the target (Figure 1a) and generates a cone of essentially uncollimated photons, while the second arrangement collimates the photon beam (Figure 1b) and uses a copper disc placed close to the tungsten discs. The photon collimator was fabricated from a 53 mm long 316 stainless‐steel tube with 1060 aluminum alloy ends, allowing for a 50 mm thick layer of Wood's metal ^33^ to fill the remaining volume, surrounding the 14 mm diameter central aperture. The aluminum tube housing with the (in‐air target) tungsten discs, the collimator, and copper disk are easily removable, which enables a fully reversable (and interchangeable) setup for preclinical photon and electron beam arrangements.

### 2.3 Target thickness

Thin tungsten discs (thickness ≈ 55 µm) were stacked to enable a range of target thicknesses. Charge output measurements were performed with an Advanced Markus ionization chamber (AMC, model 34045, PTW‐Freiburg, Freiburg, Germany, connected to an electrometer (UNIDOS Webline, PTW‐Freiburg, Freiburg, Germany, and operated at a bias voltage of +300 V), at the surface (SSD of 64 mm) and at various depth in solid water (150 × 150 mm^2^ RW3 slabs of 1, 2, 5, and, 10 mm thicknesses from PTW‐Freiburg) in the un‐collimated photon setup (Figure 1b), to experimentally determine the photon output variation with tungsten thickness, as well as the thickness required to remove primary electrons.

### 2.4 Beam current collection

In addition to the beam charge monitor described above, a beam collection device was used to measure the approximate electron beam current (without a bremsstrahlung target in place). This consisted of an insulated 46 mm diameter, 50 mm thick aluminum disc, placed inside a grounded aluminum cup placed ∼36 mm downstream of the exit window and connected (using RG58 coaxial cable) to a 50 Ω terminated at the input of a transient digitizer (Picoscope 4603, Pico Technology, St Neots, Cambridgeshire, UK). This beam collection device acted as an unbiased Faraday cup and since the collecting electrode was thick enough to stop all incoming electrons, provided an output directly proportional to the accelerator output current. Here, secondary electron production and backscatter ^34^ inevitably affect the collected current. Calibrated attenuators could be inserted in series when the peak voltage generated exceeded the capabilities of the digitizer input stage. Gun heater current was adjusted from 6 to 8A, in increments of 0.04 A, where settings were repeatable to within ± 0.02 A. This current is an indicator of electron emission from the gun, but no physical meaning can be attributed to it. With the bremsstrahlung target assembly in place, photon charge output was measured with the AMC (positioned at 10 cm depth in RW3 solid water) by delivering 30 pulses for each gun heater current setting.

### 2.5 Measurements of dose rates, beam profiles, and depth‐dose curves

Gafchromic EBT‐XD film (Ashland Inc., Covington, Kentucky, USA) were used for measurements of average dose rates, beam profiles and for generation of percentage depth‐dose (PDD) curves, with films read out 24 h post‐irradiation using a film scanner (Epson Perfection v850 Pro, Seiko Epson Corporation, Nagano, Japan) at 96 dpi. Films were analyzed (red channel) using ImageJ (v2.14.0/1.54f). Films had previously been calibrated in both 6 MV photon and 6 MeV electron clinical beams from a Varian TrueBeam (Varian Medical Systems Inc., Palo Alto, California, USA) linac at the Churchill Hospital in Oxford, United Kingdom. To eliminate scanner warm up effects, five scans were taken without films present prior to film measurements. Average dose rate measurements were performed with 35 × 35 mm^2^ films placed on the surface of the solid water phantom at various SSDs, in both the photon and electron beams (Figure S3). The average dose rates were determined within a 6.6 mm (25 pixel) diameter region of interest (ROI). For depth‐dose measurements with electrons (Figure S4) and photons, a rectangular 5 × 5 mm^2^ (19 × 19 pixels) area ROI was analyzed. For each depth‐dose measurement, the total thickness of solid water was such that at least 5 cm of solid water backscatter material was present (to closely link to TRS‐398^35^ and TRS‐483^36^ protocols). Three repeated film measurements were averaged for average dose rate and depth‐dose measurements. Beam profiles were analyzed using 80 × 80 mm^2^ films by averaging along a 5 mm (19 pixel) line perpendicular to the profile axis, for each pixel traversed across the profile axis. For vertical and horizontal measurements, two repeated measurements were averaged. Two unirradiated background films were consistently used for all films to convert the optical density value to dose.

## 3 RESULTS

### 3.1 Target thickness

**FIGURE 2:**
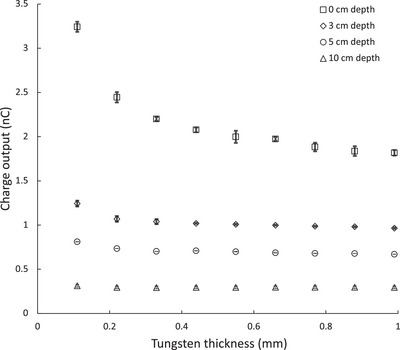
Charge output measured with the Advanced Markus ionization chamber positioned at various depth in solid water vs. Tungsten thickness using the uncollimated photon setup (Figure 1a). Markers and error bars represent the mean value and standard deviation, respectively, of five measurements.

The charge output was found to be largely independent of target thickness (Figure 2), within the investigated range (0.11–0.99 mm). However, there was a pronounced increase at the surface of the solid water for small foil thicknesses, which was most likely due to contribution from the low energy photon fluence to the collected charge (Figure S5). This effect became small for target thicknesses ≥ 0.3 mm and negligible at depths beyond the CSDA range of 6 MeV electrons in water ∼3 cm. The contribution of low energy photon components was reduced with thicker foils and with collimation (see Figures S5–S9). As mentioned previously, a thinner target also results in less temperature increase. Based on these results, a tungsten target thickness of 0.55 mm was found to be a suitable compromise and was therefore used in all subsequent measurements.

### 3.2 Photon output variation with gun heater current

**FIGURE 3:**
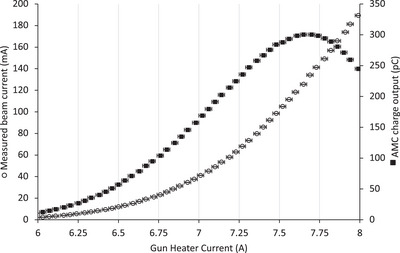
Beam currents measured with a beam collection device placed in the electron beam path (circles), and charge outputs (squares) from the bremsstrahlung target measured with the Advanced Markus ionization chamber (AMC) as a function of gun heater current. A 30‐pulse delivery was used, with the AMC placed at 10 cm depth in solid water. Error bars represent the standard deviation of five measurements.

Measurements with the beam collection device placed in the beam (replacing the bremsstrahlung target) showed how the beam current increased with increasing gun heater current (Figure 3). However, because beam loading caused a reduced beam energy at high beam currents, photon charge output was found to be maximized at a gun heater current in the range of 7.56–7.78 A (within 2% of its maximum value in this range). Using the lowest gun heater current value (7.56 A) in this range, PDD measurements (at an SSD of 72 cm) show that the produced electron beam has therapeutic (*R_80_*) and practical (*R_P_*) ranges of around 20  and 30 mm, respectively (20.4 and 29.9 mm in Figure S4). These could be considered as typical values for a standard clinical 6 MeV electron beam, though these beams use electron applicators and PDDs are commonly measured at an SSD of 100 cm. Therefore, a gun heater current value of 7.56 A was chosen for all subsequent experiment with photons.

### 3.3 Photon beam characteristics

**FIGURE 4:**
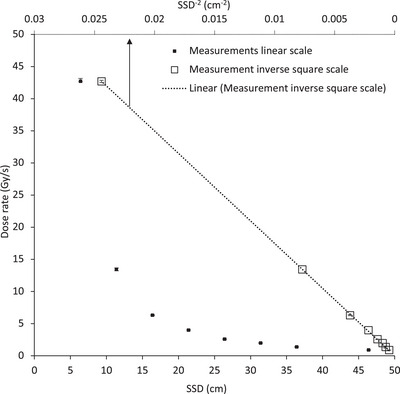
Photon surface average dose rate at different source‐to‐surface distances (SSDs), measured with EBT‐XD film on the surface of a 150 × 150 × 150 mm^3^ solid water phantom. The markers indicate the mean value of 3 repeated measurements, with error bars indicating the standard deviation. The additional horizontal axis represents the inverse square of the SSD, with a linear fit applied to the data points with an R^2^ of 0.99.

A square‐law dependence would be expected from point‐like radiation sources. Our electron beam diameter is 10 mm at the target, but we still see a good agreement with the inverse square law with an *R* ^2^ value of 0.99 (Figure 4).

**FIGURE 5:**
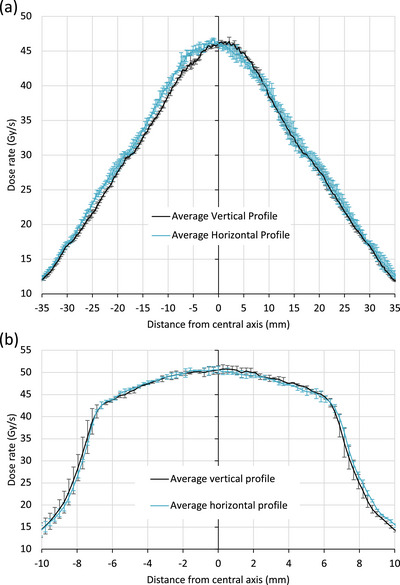
Beam profiles measured with EBT‐XD film on the surface of a 150 × 150 × 150 mm^3^ solid water phantom, at 0 mm depth for uncollimated beam (a) and with (b) 14 mm collimated beam. The markers indicate the mean value of 3 repeated measurements, with error bars indicating the standard deviation.

Beam profile measurements show that without collimation the beam profile is similar to an FFF beam (Figure 5a), with a fairly flat (≥97% of the maximum dose) central 10 mm diameter section and 90% dose relative to the maximum of the profile within 15 mm diameter of the circular beam. With collimation, the surface dose rate increases slightly and the flatness is similar, with 90% dose relative to the maximum of the profile within 12 mm diameter of the circular beam. At larger distances from the beam center, the resulting beam penumbra from the collimation is evident (Figure 5b).

**FIGURE 6:**
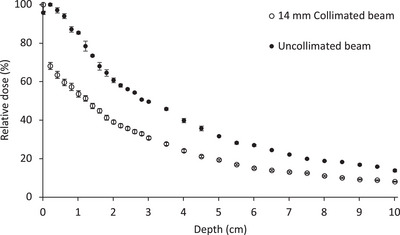
Depth‐dose curves measured with EBT‐XD film at various depths in a 150 × 150 × 150 mm^3^ solid water phantom, for the uncollimated beam as well as the 14 mm collimated beam. The markers indicate the mean value of 3 repeated measurements, with error bars indicating the standard deviation.

**FIGURE 7:**
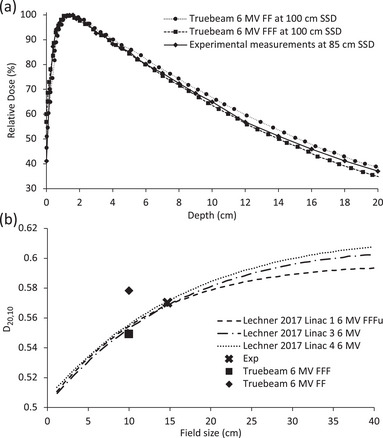
(a) Percentage depth‐dose curves; measured with EBT‐XD film at various depths in a 150 × 150 × 250 mm^3^ solid water phantom, at a source‐to‐surface distance (SSD) of 85 cm, for the 14 mm collimated beam, which expands to 16.3 cm FWHM at the surface of the phantom. The markers indicate the mean value of three repeated measurements, with error bars indicating the standard deviation. Also included are published curves for 10 × 10 cm^2^ fields from a 6 MV TrueBeam at an SSD of 100 cm, with (FF) and without flattening filter (FFF).^37^ (b) Published ratio of doses at depths of 20 and 10 cm (D_20,10_) ^37^, ^38^ for 6 MV FFF beams with various field sizes, with points added from measured and published doses in (a). The measurement field size was multiplied by a correction factor of 0.9 to convert from a circular to a square field size to be 14.7 cm, with a D_20,10_ value of 0.57.

Depth‐dose measurements showed an increased dose rate at the surface of the solid water phantom from scattered photons (Figure 6), particularly for the collimated beam, where a decrease in dose rate occurs with depth mainly due to the inverse square law (Figure 4), as the SSD is short (64 mm). For a long SSD of 85 cm, the measured depth‐dose curve is similar to depth‐dose curves from clinical 6 MV (nominal) beams from a Varian TrueBeam ^37^ at 100 cm SSD, with or without flattening filter (Figure 7a). These depth‐dose curves were further characterized using the ratio of doses at depths of 20 and 10 cm (D_20.10_) in solid water to show further agreement with published beam data (Figure 7b).

## 4 DISCUSSION

Photon FLASH radiotherapy has shown potential with kilovoltage (kV) continuous (non‐pulsed) beams.^39^, ^40^ Unfortunately, such beams have large surface average dose rates which drop steeply from the surface and enable delivery of FLASH dose rates at depths of only up to a few mm. To reach ultra‐high average dose rates, the SSD is short which limits beam size, for example, with a uniform beam over a ∼1 cm diameter, for an SSD of ∼3.5 cm.^41^ Several preclinical studies have also indicated the potential of photon FLASH radiotherapy to spare normal tissue at MV energies.^42^, ^43^, ^44^, ^45^ Photons at these energies can target deep seated tumors by depositing energy at larger depths, with less surface dose. The average dose rates and distributions from kV and MV photon beams could also be improved using multiple beams from different directions simultaneously.^39^, ^46^

At the Chengdu Terahertz Free Electron Laser facility (CTFEL), a large superconducting linac has been shown to produce 6–8 MeV electrons with average currents up to 10 mA, enabling photon average dose rates of > 1000 Gy/s, albeit with a significantly different pulse structure (no macropulses) than clinical linacs. Shi et al. used the CTFEL for preclinical FLASH studies with 5 ps micro pulses at 54 MHz, with instantaneous photon dose rates of > 1.8 × 10^5^ Gy/s, and an average dose rate between 110 and 120 Gy/s.^47^ Here, FLASH normal tissue‐sparing effects were seen in mice following abdominal irradiation, and in intestinal crypt organoid irradiations. However, another facility with beam parameters such as total dose, pulse structure, field size, and beam energy different than the CTFEL beam was used for the conventional average dose rate irradiations. This makes it difficult to draw any strong conclusions from these preclinical experiments.

The work described in this article enables delivery of pulsed beams of varying ultra‐high average dose rates that are appropriate for routine preclinical FLASH experiments with MV photons. Albeit, on the low end of the average dose rates that have exhibited a FLASH effect (> 40  and > 30 Gy/s from surface to ∼8 mm depth, for the uncollimated and collimated beam respectively) with similar beam parameters.^1^ This system also has the advantage of being able to perform preclinical FLASH experiments with varying average dose rates from ultra‐high down to conventional (∼0.1 Gy/s), by varying the pulse repetition rate and/or macropulse amplitude [by adjusting the heater gun current while slightly (de)tuning the RF to maintain the beam energy ^31^], with either MV photons or MeV electron beams. With our system, a direct comparison between these modalities (with identical pulse structures) is possible, which is invaluable to understand the applicability of previous MeV electron preclinical FLASH data to clinical MV photon beams—arguably the modality of interest for clinical translation of FLASH radiotherapy. Meanwhile, the limited field size (∼12–15 mm diameter) is akin to the kV‐systems described above, but still useful for a range of biological assays involving thin samples to avoid a large average dose rate falloff with depth. We have described easily reversible modifications using an in‐air target system that can be applied to linacs with similar beam parameters, for example, clinical linacs modified for electron FLASH delivery. For accuracy, repeatability and reproducibility, we followed the recommendations of dosimetric reporting standards, ^48^, ^49^, ^50^, ^51^ where we obtained results using reproducible dosimetric procedures based on primary dosimetric standards with a fully defined experimental setup. Here we stated the average dose rate, distance from source, field size at surface, filtering systems, flatness, shape, and symmetry with high resolution, with low uncertainties for repeated measurements. The guidance from TG‐51,^52^ TRS‐398,^35^ and TRS‐483^36^ were implemented in so far as their scope pertained in preclinical contexts, where field size and SSD are reduced relative to clinical beams. Furthermore, we highlight our beam pulse structure, as it is likely to impact biological outcome in preclinical FLASH studies.^53^

The dose rate efficiency of our beam is ∼0.05% (comparing average dose rates in Figures 5 and S3). To increase this efficiency, the photon dose rate may be increased slightly by decreasing the copper thickness, for example, from 6 to 3 mm. However, this would increase the magnitude of contaminant electron dose (Figure S10). Our current linac is limited by the peak power generated by our 2 MW magnetron; other linacs, fitted with higher peak/average power radiofrequency sources or with more efficient accelerating waveguide designs, would likely allow for photon beams to be produced with even higher dose rates.

Photon targets optimized through simulation by Geant4,^54^ FLUKA, ^55^ MCNP6,^28^ and other techniques ^56^ for use with 6 MeV electron beams have been published. Such targets are intended for continuous operation rather than short operation times associated with FLASH irradiations. Nevertheless, these studies show that a combination of materials (such as tungsten and copper) are generally superior to a single‐material target. It is noted that targets for continuous work in electron linear accelerators are subjected to high‐frequency and intense thermal shocks. Elevated temperatures in the target may lead to target re‐crystallization, fatigue cracking, creep and vaporization.^57^ Tungsten target thicknesses for optimal photon production have often been found to be ∼$0.3\;R_{CSDA}.$ ^54^, ^55^, ^58^, ^59^, ^60^ In this work, we used an experimental approach and found it straightforward to reach an average dose rate appropriate for preclinical FLASH work. Further target optimization is the subject of ongoing work.

## 5 CONCLUSION

We have presented an approach that can be used to perform preclinical FLASH irradiation studies with 6 MV photons. The resulting pulsed beam characteristics show that the dose rates (average and instantaneous) are high at surface but reduce quickly with depth because of the SSD used, which also limits field size and flatness. Currently, the resulting beam is only suitable for irradiation of small, thin in vitro samples and for superficial in vivo (mice) irradiations, for example, investigating the FLASH effect for skin toxicity or subcutaneous tumors, where there are no stringent requirements for high conformality, and the relatively large beam penumbra will not markedly affect outcomes. We are currently investigating means of optimizing the target design through simulations, rather than with an experimental approach.

Cancer Research UK—RadNet (C6078/A28736) financial support is gratefully acknowledged. The authors thank the Medical Research Council for financial support through a Programme grant (MR/X006611/1) and for funding ERJFT studentship. They also thank the National Cancer Institute/NIH/DHHS for their support towards this work (1P01CA257904).

## CONFLICT OF INTEREST STATEMENT

The authors have no conflicts to disclose

## Supporting information



Supporting Information

## Data Availability

Authors will share data upon request to the corresponding author.
