# Supplementary material for: Megavoltage photon FLASH for preclinical experiments
Source: Med Phys. 2025 May 19;52(7):e17891. doi: 10.1002/mp.17891 (PMC12258002; doi:10.1002/mp.17891)
Supplement: Supplementary file 1 — Supporting Information [file MP-52-0-s001.docx]

# Supporting Material

**1. Planar fluence electron energy distribution at upstream surface of tungsten converter material**


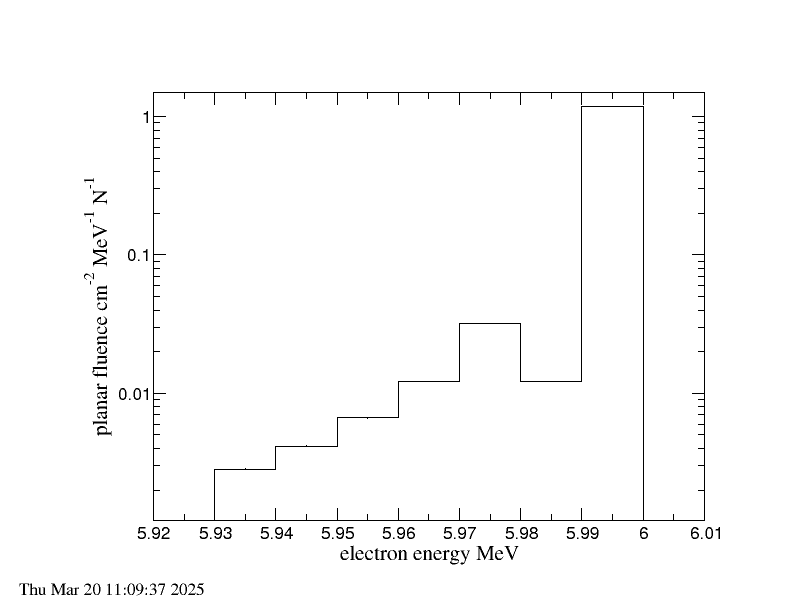


*Figure S1: EGSnrc simulation of the planar fluence energy distribution of electron beam incident on the upstream surface of the tungsten disc. A monoenergetic 6 MeV beam of 1e7 particles was modelled passing through the beryllium copper exit window and air separation before reaching the upstream tungsten surface.*

**2. Electron beam profile at target position**

*Figure S2: Electron beam profile measurement with a 25 mm diameter EBT-XD film placed on the upstream surface of the tungsten discs, within the bremsstrahlung target assembly.*

**3. Variation of electron surface dose rate with source-to-surface distance**

*Figure S3: Electron surface dose rate in kGy/s at different source-to-surface distances, measured with EBT-XD film on the surface of a 150 x 150 x 150 mm^3^ solid water phantom. The markers indicate the mean value of 3 repeated measurements, with (small and within the marker) error bars indicating the standard deviation.*

**4. Electron beam Percentage Depth Dose curve**

*Figure S4: Percentage depth-dose curves for our 6 MeV (nominal) electron beam, measured with EBT-XD film at various depth in a 150 x 150 x 150 mm^3^ solid water phantom at a source-to-surface distance of 72 cm, for a heater gun current of 7.57 A. The markers indicate the mean value of 3 repeated measurements, with error bars indicating the standard deviation.*


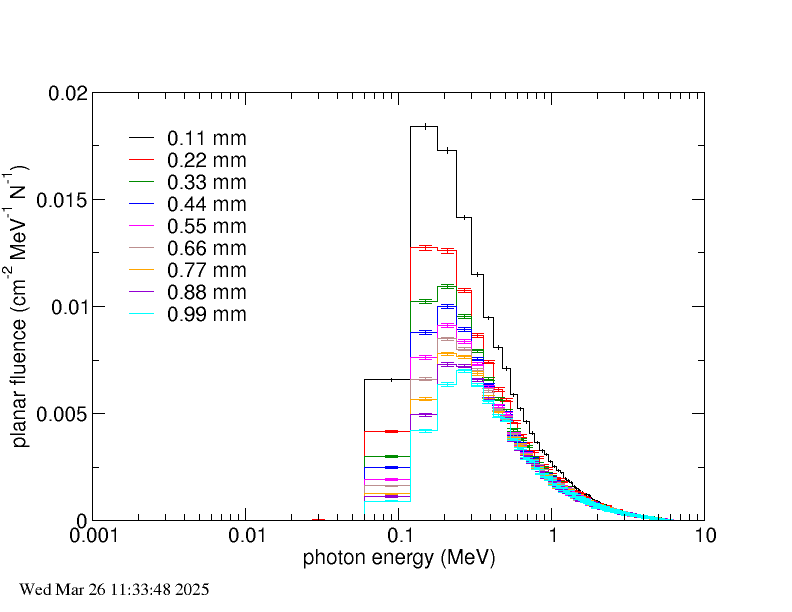
**5. Planar fluence photon energy distribution in the uncollimated arrangement with varying tungsten disc thickness**

*Figure S5: EGSnrc simulation of the planar fluence energy distribution of photons reaching the surface of the solid water slabs. A monoenergetic 6 MeV beam of 1e6 particles was incident on varying tungsten discs of thicknesses 0.11-0.99 mm with a 6 mm copper hardening filter in the uncollimated arrangement in figure 1A.*

**6. Distribution of particle energies contributing to dose**

*Figure S6: Geant4 simulation of the energy distribution of particles contributing to dose at 1 mm depth in water. A 5.0 mm diameter, 1 mm thick cylindrical water scoring volume was used for uncollimated and 14 mm collimated arrangements with a 0.55 mm tungsten disc and 6 mm copper hardening filter.*

**7. Planar fluence photon energy distribution in the collimated arrangement with varying tungsten disc thickness**

**
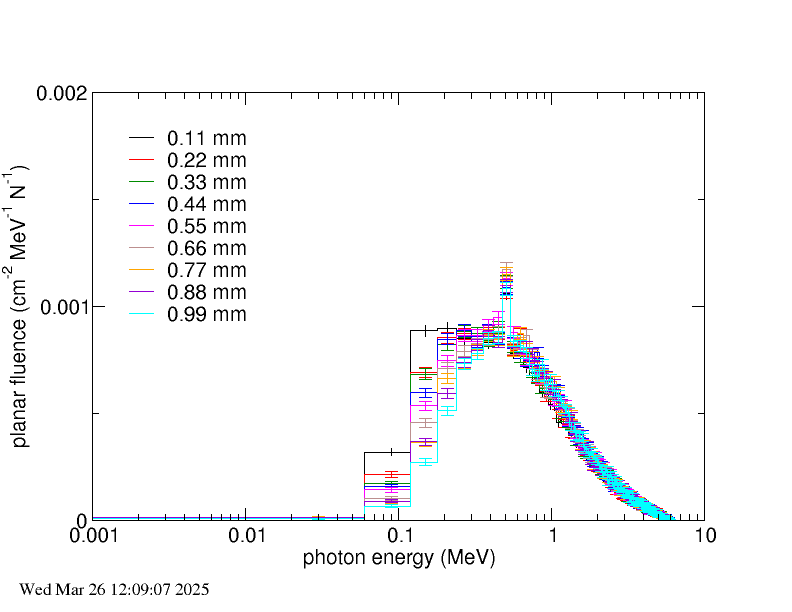
**

*Figure S7: EGSnrc simulation of the planar fluence energy distribution of photons reaching the surface of the solid water slabs. A monoenergetic 6 MeV beam of 1e6 particles was incident on varying tungsten discs of thicknesses 0.11-0.99 mm with a 6 mm copper hardening filter in the collimated arrangement in figure 1B.*

**8. Planar fluence photon energy distribution in the collimated arrangement with varying copper hardening filter thickness
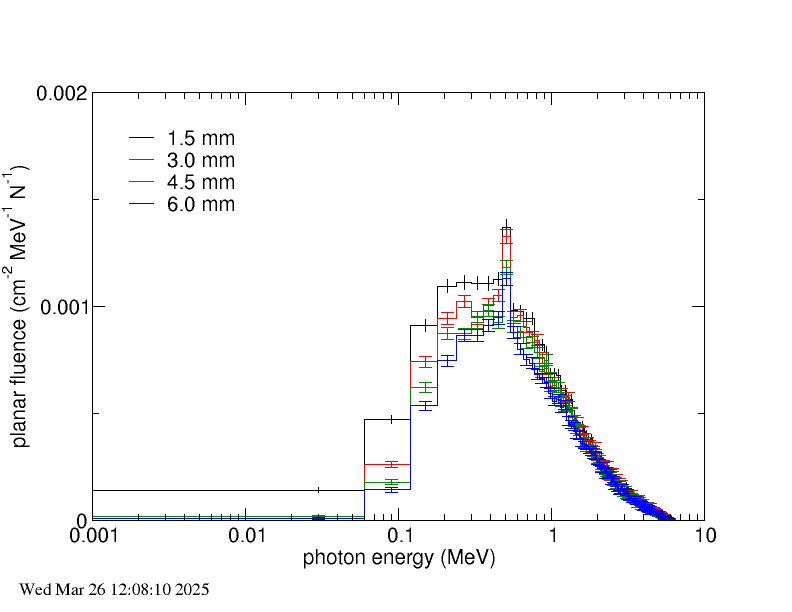
**

*F**igure S8: EGSnrc simulation of the planar fluence energy distribution of photons reaching the surface of the solid water slabs. A monoenergetic 6 MeV beam of 1e6 particles was incident on 0.55 mm tungsten disc with varying copper hardening filter of thicknesses 1.5-6.0 mm in the collimated arrangement in figure 1B.*

**9. Planar fluence photon energy distribution in the uncollimated arrangement with varying copper hardening filter disc thickness
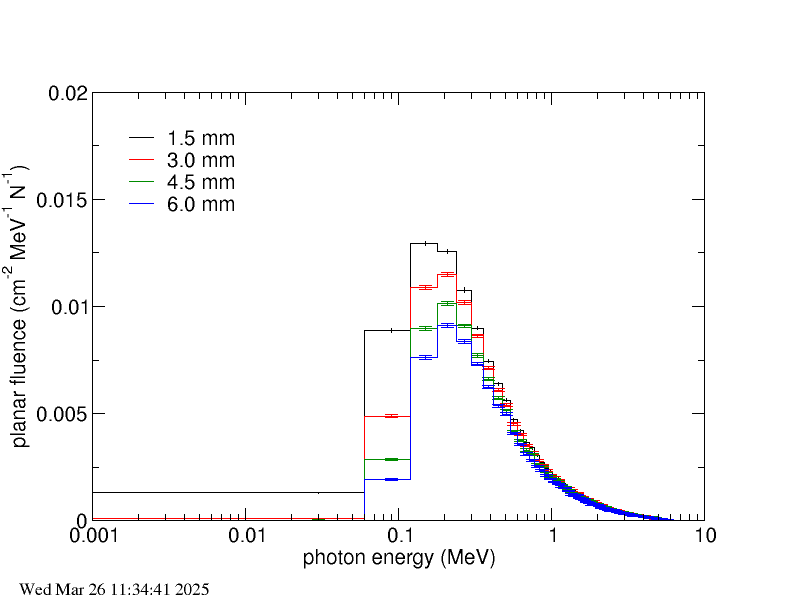
**

*Figure S9: EGSnrc simulation of the planar fluence energy distribution of photons reaching the surface of the solid water slabs. A monoenergetic 6 MeV beam of 1e6 particles was incident on a 0.55 mm tungsten disc with varying copper hardening filter of thicknesses 1.5-6.0 mm in the uncollimated arrangement in figure 1A.*

**10. Primary electron dose contamination for uncollimated and collimated arrangements**

*Figure S10: Geant4 simulation of the primary electron dose contamination percentage at 1 mm depth in a 15x15x15 cm^3^ water block, for a 5 mm diameter, 1 mm thick sensitive water volume. A monoenergetic 6 MeV beam of 1e7 particles was incident on a 0.55 mm tungsten disc with varying copper hardening filter of thicknesses 1.5-7.5 mm in the uncollimated and collimated arrangements in figure 1A and 1B.*
